# Targeting of Voltage-Gated Calcium Channel α_2_δ-1 Subunit to Lipid Rafts Is Independent from a GPI-Anchoring Motif

**DOI:** 10.1371/journal.pone.0019802

**Published:** 2011-06-10

**Authors:** Philip Robinson, Sarah Etheridge, Lele Song, Riddhi Shah, Elizabeth M. Fitzgerald, Owen T. Jones

**Affiliations:** Faculty of Life Sciences,University of Manchester,Core Technology Facility, Manchester, United Kingdom; University of California, Berkeley, United States of America

## Abstract

Voltage-gated calcium channels (Ca_v_) exist as heteromultimers comprising a pore-forming α_1_ with accessory β and α_2_δ subunits which modify channel trafficking and function. We previously showed that α_2_δ-1 (and likely the other mammalian α_2_δ isoforms - α_2_δ-2, 3 and 4) is required for targeting Ca_v_s to lipid rafts, although the mechanism remains unclear. Whilst originally understood to have a classical type I transmembrane (TM) topology, recent evidence suggests the α_2_δ subunit contains a glycosylphosphatidylinositol (GPI)-anchor that mediates its association with lipid rafts. To test this notion, we have used a strategy based on the expression of chimera, where the reported GPI-anchoring sequences in the gabapentinoid-sensitive α_2_δ-1 subunit have been substituted with those of a functionally inert Type I TM-spanning protein – PIN-G. Using imaging, electrophysiology and biochemistry, we find that lipid raft association of PIN-α_2_δ is unaffected by substitution of the GPI motif with the TM domain of PIN-G. Moreover, the presence of the GPI motif alone is not sufficient for raft localisation, suggesting that upstream residues are required. GPI-anchoring is susceptible to phosphatidylinositol-phospholipase C (PI-PLC) cleavage. However, whilst raft localisation of PIN-α_2_δ is disrupted by PI-PLC treatment, this is assay-dependent and non-specific effects of PI-PLC are observed on the distribution of the endogenous raft marker, caveolin, but not flotillin. Taken together, these data are most consistent with a model where α_2_δ-1 retains its type I transmembrane topology and its targeting to lipid rafts is governed by sequences upstream of the putative GPI anchor, that promote protein-protein, rather than lipid-lipid interactions.

## Introduction

Voltage-gated calcium channels (Ca_v_s) represent the primary means by which changes in membrane potential are coupled to the influx of second messenger calcium ions [1]. As such, Ca_v_s play a major role in orchestrating diverse excitable cell functions, ranging from rapid events such as neurotransmitter release in nerves and excitation-contraction coupling in muscle, to longer lasting events such as synaptic plasticity. While it is well established that disruption of Ca_v_s is involved in diverse pathologies, including neuropathic pain [2] and cardiac arrhythmia [3], much less is known about how Ca_v_ functionality is modulated, physiologically, at the cellular level [4].

Biochemical and reconstitution studies show that Ca_v_s comprise an α_1_ subunit (≈200 kDa) containing the voltage-sensing, gating and pore machineries [1], [5]. In high voltage-activated Ca_v_1 and Ca_v_2 family channels, α_1_ is complexed in a 1∶1 stoichiometry with a cytoplasmic auxiliary β subunit. These channels are also complexed with a second auxiliary (≈125 kDa) subunit termed α_2_/δ, which, like β subunits, enhances cell surface expression and modulates the biophysical properties of channel heteromers [1], [6], [7]. Since multiple genes encode each type of Ca_v_ subunit and their transcripts undergo RNA splicing, Ca_v_s manifest a considerable potential for diversity not only in terms of biophysical function, but also in their modulation and cellular expression patterns [1], [7].

Irrespective of their location, emerging data has shown that Ca_v_s are organised into large heterogeneous macromolecular assemblies containing a plethora of signal transduction proteins with which they interact and co-operate to meet local and global functional demands [4], [8], [9], [10]. Defining the mechanisms by which such assemblies are constructed and distributed is therefore crucial to understanding and manipulating Ca_v_ function [10], [11], [12]. In this regard, an important step forward has been the observation that Ca_v_ proteins co-localise with components of specialised cholesterol-rich membrane signalling domains termed lipid rafts [13], [14], in both heterologous expression systems and native tissues [15]–[21]. While alterations in Ca_v_ currents seen with cholesterol-depleting agents argue that raft-association is physiologically significant, the precise effects appear to be subtype and/or tissue specific [16], [18]–[21]. Although different Ca_v_s may associate with rafts using alternate modalities [18], [22], there is now compelling evidence for a major involvement of the α_2_/δ subunit [18], [20], [21]. Thus, α_2_/δ subunits co-localise with the lipid raft marker proteins caveolin and flotillin when expressed alone [18], [20], [21] and are also necessary and sufficient for the targeting of Ca_v_2.2 complexes to rafts [21].

**Figure 1:**
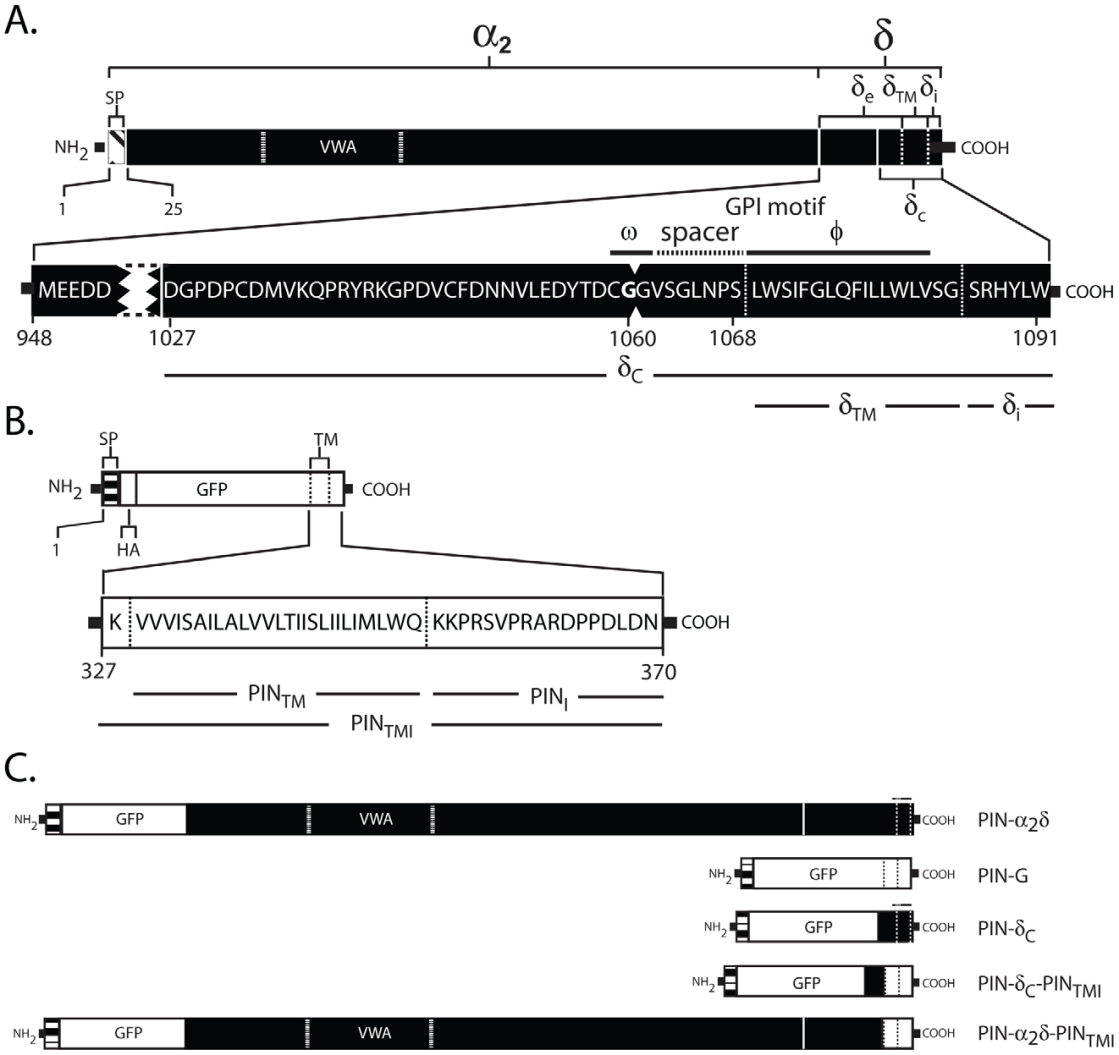
Depiction of WT α_2_/δ-1 (A), the PIN-G reporter (Genbank AY841887.1) [**51**] (B) and chimeric (C) constructs. Throughout, numbering is based on the full-length polypeptide prior to signal peptide cleavage and cartoons for all constructs are approximately to scale. A. Wild type α_2_/δ showing positions of the α_2_ and δ polypeptides and Von Willebrand factor A (VWA) domain. In α_2_/δ-1, residues 1–25 encode the signal peptide (SP). The δ subunit is further subdivided into exofacial (δ_e_), putative transmembrane (δ_TM_) and intracellular (δ_i_) regions. The putative minimal GPI anchoring motif, located within a cysteine-rich region (δ_c_) proximal to the external face of the lipid bilayer, contains, in turn, the ω residue (Gly1060) to which GPI is attached, a short spacer (dashed line) and a largely hydrophobic region. Indents between residues 1060 and 1061 indicate chimera fusion site where all downstream δ sequences in constructs PIN-δ_c_ or PIN-α_2_/δ were replaced by the transmembrane and intracellular carboxy terminal residues of PIN-G (constructs PIN-δ_c_-PIN_TMI_ and PIN-α_2_/δ-PIN_TMI_). The parent construct PIN-G (B) contains a signal peptide derived from the Igκ chain, an exofacial haemagglutin (HA) epitope tag, green fluorescent protein (GFP) a carboxy terminal sequence (PIN_TMI_) containing the transmembrane spanning domain from the platelet-derived growth factor receptor and a 17 residue intracellular inert region, whose modification with endocytic or other cytoplasmically exposed targeting motifs can be used to re-direct the reporter to specific intracellular organelles [51]. Chimera (C) include PIN-δ_c_, where the entire transmembrane and intracellular region (Residues 327–370) of PIN-G was replaced by WT α_2_/δ residues 1027–1091 (i.e. δ_c_, δ_TM_, δ_i_); PIN-δ_c_-PIN_TMI_ corresponding to a PIN-G construct containing δ_c_ residues (1027–1060) inserted prior to the PIN_TMI_ region. Additional chimera include PIN-α_2_/δ, corresponding to exofacial PIN-G residues 1–326 fused to the amino terminus of WT α_2_/δ-1, and PIN-α_2_/δ-PIN_TMI_, where the C-terminal residues (WT α_2_/δ-1: 1061–1091 (see A)) were replaced by the entire transmembrane and intracellular region (Residues 327–370) of PIN-G. While the putative GPI anchoring motif (lines in C) is present in PIN-δ_c_ and PIN-α_2_/δ, it is absent in PIN-G and disrupted in PIN-δ_c_-PIN_TMI_ and PIN-α_2_/δ-PIN_TMI_ chimera. Vertical solid and dashed lines denote α_2_/δ and transmembrane domain boundaries, respectively.

Until recently, how the α_2_/δ subunit might mediate Ca_v_ raft targeting was unclear. Structurally, the α_2_/δ subunit has been viewed as a type I transmembrane (TM) spanning protein (Fig. 1A) composed of a large exofacial α_2_ head region linked via disulfide bonds to a smaller membrane associated δ subunit [1], [7], [23], [24], [25]. Owing to the presence of features such as Von Willebrand factor A (VWA) and Cache domains, commonly found in integrins and other cell surface proteins, the α_2_ region is thought to have a modular structure [6], [7], [26] affording interactions with extracellular matrix proteins such as thrombospondin [27]. Structure-function analysis has also shown that the α_2_ region mediates those interactions with Ca_v_s that support current enhancement and the biophysical effects seen upon co-expression of α_2_/δ subunits with α_1_/β complexes [28], [29]. In contrast, the δ polypeptide, while affecting the voltage-dependence of Ca_v_s [28], has been viewed as primarily providing a means for attaching the α_2_ polypeptide to the cell surface via its hydrophobic putative TM-spanning domain located proximal to the short, intracellular, carboxy terminus [1], [7], [20] [23]–[25]. However, a recent study has challenged this structural model and offered a new mechanism for Ca_v_ raft localisation by suggesting the α_2_/δ subunit associates with the plasma membrane via a glycosylphosphatidylinositol (GPI) anchor attached to the δ polypeptide [20]. In common with other GPI anchored proteins, GPI attachment is envisaged to occur through the action of an ER-resident GPI-transamidase which recognises, cleaves and modifies a motif located at the distal carboxy terminus [28], [30]–[32]. While such anchoring motifs do not have a strict consensus sequence, they contain common elements including a) an amino acid with a small side chain (notably G, C, D, A, N or S) known as the ω site/residue, to which the GPI moiety is amide-linked, b) two adjacent residues (ω+1..2) with small side chains (typically G, A and S), c) a spacer sequence of >6 hydrophilic residues, commencing at the ω+3 position and d) a stretch of hydrophobic residues (particularly L) capable of spanning the membrane [30], [31].

Since GPI-anchored proteins are highly concentrated in lipid rafts [14], [33], [34], the revised model of α_2_/δ subunit structure has then been used to rationalise Ca_v_ raft targeting [20], [35] and the apparent weakness of the α_2_/δ subunit-α_1_/β complex interaction [10], [36]. However, while seeming attractive in offering GPI attachment as a further regulatory locus [35], such a model requires that lipid-lipid interactions between a single δ subunit GPI anchor and liquid-ordered (L_o_) raft lipids [37] can specify the raft association of Ca_v_α_1_ (+β), a large, multispanning, membrane protein complex, predicted to partition into liquid-disordered (L_D_), bulk phase lipid [14], [38], [39]. Moreover, of the four mammalian α_2_/δ subunits, only α_2_/δ-3 shows a significant potential for GPI anchoring when analysed by predictive algorithms (Table 1). Recent evidence also indicates that the co-localisation of raft markers and α_2_/δ-1 subunits (when expressed alone or with α_1_/β complexes) in cell surface aggregates demands an intact actin-based cytoskeleton [21]. However, while this is consistent with a role for actin in shaping the distribution and dynamics of GPI-anchored proteins [40], [41], [42], such observations are equally consistent with the hypothesis that α_2_/δ-1 subunits reside in rafts, and/or higher order raft assemblies, via organising principles based upon protein-protein [21], [42], [43], [44] and/or specialised lipid-protein [14], [45], [46], [47] interactions.

**Table 1:** Comparison of predicted GPI-anchoring potential for WT α_2_δ-1, PIN-α_2_δ chimera, mutant α_2_δ-2 GAS:WKW and Thy-1.

Protein	BIG-PI	FragAnchor	PredGPI
WT α_2_δ-1	No (−26.06; P = 0.039; CG**G**V)	Rejected (NN 0.491)	Probable (CGG**V** )
WT α_2_δ-2	No (−46.37; P = 0.2; G**A**SF)	Rejected (NN 2.8×10^−5^)	Not GPI-anchored
WT α_2_δ-3	Yes (+8.86; P = 3.1×10^−4^; E**C**GG)	Accepted (NN 0.999)	Highly probable ( **E**CGG)
WT α_2_δ-4	No (−7.03; P = 4.8×10^−3^; **N**AQD)	Probable (NN 0.989)	Probable (D**C**GG)
PIN-G	No (−85.82; P = 0.87; R**S**VP)	Rejected (NN 3×10^−6^)	Not GPI-anchored
PIN-α_2_δ	No (−26.06; P = 0.039; CG**G**V)	Rejected (NN 0.491)	Probable (CGG**V** )
PIN-α_2_δ -PIN_TMI_	No(−85.82; P = 0.87; R**S**VP)	Rejected (NN 3×10^−6^)	Not GPI-anchored
PIN-δ_C_	No (−26.06; P = 0.04; CG**G**V	Rejected (NN 0.491)	Probable (CGG**V** )
PIN-δ_C_-PIN_TMI_	No (−85.82; P = 0.87;R**S**VP)	Rejected (NN 3×10^−6^)	Not GPI-anchored
α_2_δ-2 GAS:WKW	No (−51.73; P = 0.28; P**S**LG)	Rejected (NN 1.1×10^−5^)	Not GPI-anchored
Thy-1	Yes (+11.44; P = 1.7×10^−4^; **C**GGI)	Accepted (NN 0.999)	Highly probable ( **C**GGI)
GFP-GPI	Yes (+11.75; P = 1.57×10^−4^; AM**S**G)	Accepted (NN 0.999)	Highly probable (AM**S**G)

Proteins were analysed using three independent algorithms Big-PI [30] (http://mendel.imp.ac.at/gpi/gpi_server.html), FragAnchor [55] (http://navet.ics.hawaii.edu/~fraganchor/NNHMM/NNHMM.html) and PredGPI [56] http://gpcr.biocomp.unibo.it/predgpi/). Big-PI is a predictor based on scoring the presence of an amino terminal signal peptide and features of canonical carboxy-terminal GPI-anchoring motifs. FragAnchor identifies GPI motifs using a Neural Network (NN) and Hidden Markov Model (HMM). PredGPI integrates a Support Vector Machine and HMM and employs accurately trained datasets. Likelihood of GPI anchoring is indicated by positive scores in Big-PI, NN values ≈1 in Frag Anchor and a ranking (Highly probable, probable, lowly probable and not GPI-anchored) in PredGPI. Of the three algorithms only Big-PI and PredGPI predict ω-site residues (bold and underlined in tetrapeptide sequences indicated), with the latter reported to afford the lowest rate of false positive predictions. Note that the ω-site residues giving the highest potential for GPI-modification are indicated, irrespective of the protein's potential for GPI modification. While differences exist in the predicted ω-site residues obtained between algorithms, these are generally in very close physical proximity. Of the four WT Ca_v_-α_2_δ subunits only α_2_δ-3 is predicted to be GPI-anchored by all three algorithms while WT α_2_δ-1 is only predicted to be, using PredGPI. In addition, the predicted ω-site for WT α_2_δ-1 differs between algorithms (Big-Pi: CG**G**V; PredGPI: CGG**V** ) and also to that reported [20](C**G**GV).

To resolve the above hypotheses we have re-visited the raft localisation of the α_2_/δ subunit using an established strategy [48], [49], [50] based on the expression of chimera, where the reported GPI-anchoring sequences in α_2_/δ-1 have been swapped with those from a known Type I TM-spanning protein – PIN-G (Fig. 1B) [51]. Like its α_2_/δ-2 and α_2_/δ-3 counterparts, α_2_/δ-1 has been described as a GPI-anchored protein [20]. However, unlike α_2_/δ-2 and α_2_/δ-3, the consequences of mutating the presumptive GPI-anchoring motif on α_2_/δ-1 raft localisation or Ca_v_ currents have not been reported. Using imaging, electrophysiological and biochemical assays that we recently employed to analyse α_2_/δ-1 in rafts [21], we now show that the raft localisation of α_2_/δ-1 is preserved even after replacement of the reported GPI anchoring motif with the TM domain of PIN-G. Conversely, the GPI-anchoring motif is not sufficient to target PIN-G to lipid rafts. While the localisation of a PIN construct containing α_2_/δ-1, and its GPI motif, to lipid rafts shows susceptibility to GPI-cleavage using phosphoinositide-specific phospholipase C (PI-PLC), this effect is assay-dependent and seems to lack specificity as it also disrupts the raft localisation of caveolin, but, interestingly, not flotillin. Our data therefore support a model where the raft localisation of α_2_/δ-1 depends upon exofacial sequences upstream and independent of the putative GPI-anchoring motif.

## Results

### Construction and GPI-anchoring potential of α_2_/δ-1/PIN-G chimera

To dissect the role of GPI anchoring in localising α_2_/δ subunits to lipid rafts, a series of chimera were prepared between rat α_2_/δ-1 and PIN-G, a functionally inert Type I TM protein reporter that lacks trafficking or post-translational modification motifs [51] (Fig. 1). Initially, we made a PIN chimera −PIN-α_2_/δ - encoding the PIN ‘head’ region (i.e. signal peptide, Haemagglutin (HA) and Green fluorescent protein (GFP) tags, lacking TM and intracellular domains) fused to full length α_2_/δ-1. Next, a chimera −PIN-δ_c_ - was generated by fusing the PIN head to the distal carboxy terminal region of the δ-1 polypeptide to yield a construct containing the entire purported GPI-anchoring motif of α_2_/δ-1, plus 33 residues upstream, and all residues downstream of the ω site (Wild type (WT) α_2_/δ-1∶Gly1060, [20]. Two additional constructs −PIN-δ_c_-PIN_TMI_ and PIN-α_2_/δ-PIN_TMI_ – were then designed where the putative GPI anchoring motifs within PIN-δ_c_ and PIN-α_2_/δ were disrupted by replacement of all δ residues after the ω residue, with those encoding the transmembrane and intracellular region (‘TMI’ residues 327–370) of PIN-G. Based upon the work of Davies et al., 2010 [20] both the PIN-α_2_/δ and PIN-δ_c_ constructs are predicted to be GPI anchored by virtue of the presence of the purported α_2_/δ-1 GPI-anchoring motif. However, this prediction is supported by only one (Pred-GPI) of the three independent algorithms [30], [52], [53] we employed, and even then with GPI-attachment at a different ω residue to that predicted by Davies et al., [20] (Table 1). In contrast, all three algorithms predict that PIN-G, PIN-δ_c_-PIN_TMI_ and PIN- α_2_/δ-PIN_TMI_ are not GPI-anchored (Table 1), whereas GFP-GPI, which contains the GPI-anchoring motif of the folate receptor [54] is GPI-anchored.

### The biophysical properties of PIN-α_2_/δ are retained following substitution of the GPI-anchoring motif with the transmembrane and intracellular sequence of PIN-G

**Figure 2:**
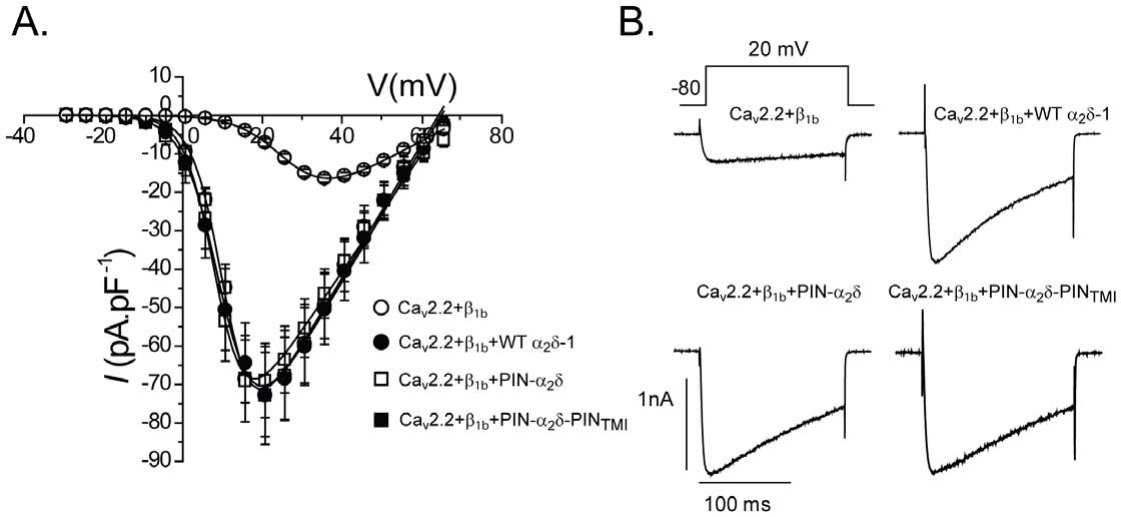
Effect of WT α_2_δ-1, PIN-α_2_δ and PIN-α_2_δ-PIN_TMI_ on Ca_v_2.2/β_1b_ currents. *(A)*Average current density-voltage (*I-V)* plots for Ca_v_2.2/β_1b_ currents in the absence of α_2_/δ-1 (open circle) and in the presence of WT α_2_/δ-1 (closed circle), PIN-α_2_δ (open square) and PIN-α_2_δ-PIN_TMI_ (closed square). Continuous lines indicate the Boltzmann fits to *I-V* plots using the function described in the Methods. *(B)* Representative peak current traces from cells expressing Ca_v_2.2/β_1b_ in the absence of α_2_δ-1 and Ca_v_2.2/β_1b_ co-expressed with WT α_2_δ-1, PIN-α_2_δ and PIN-α_2_δ-PIN_TMI_. Currents were evoked using 150 ms depolarising steps in 5 mV intervals (−30 to +65 mV), from a holding potential, *V*_h_, −80 mV. Data are shown as the mean ± S.E.M.

In order to confirm that PIN-α_2_δ was fully functional we compared its effects on the electrophysiological properties of Ca_v_2.2/β_1b_ channels, with those of WT α_2_δ-1. Preliminary experiments indicated that the presence of the GFP-tag on PIN-α_2_δ caused a marked hyperpolarisation of the *V*_50_ for activation and a slowing of both current activation and inactivation (Fig. S1). These effects are consistent with previous reports on the biophysical effects of amino-terminal modifications of the α_2_/δ subunit [55]. As a result, all subsequent electrophysiological experiments were conducted using constructs that lacked the GFP tag (deGFP; Fig. S1). As shown in Fig. 2, co-expression of PIN-α_2_δ conferred on Ca_v_2.2/β_1b_ currents the typical hallmarks associated with the presence of WT α_2_δ-1. Thus, compared with Ca_v_2.2/β_1b_ in the absence of α_2_δ-1, the peak current density, *I*_max_, was enhanced approximately 4-fold, the *V*_50_ for activation was hyperpolarised by some 13 mV on average and the rate of current inactivation was enhanced (decreased *T*_inact_) upon co-expression of PIN-α_2_δ (see also Table S1). We next examined the functional effects of disrupting the GPI anchoring motif within α_2_δ. Somewhat surprisingly, and in contrast to data for the α_2_δ-2 and α_2_δ-3 GPI-anchoring-deficient mutants [20], co-expression of PIN-α_2_/δ-PIN_TMI_ with Ca_v_2.2/β_1b_ produced identical currents to those of channels containing either PIN-α_2_δ or WT α_2_δ-1. In the absence of any α_2_ sequences there was no functional effect on Ca_v_2.2/β_1b_ channels (Table S1; PIN-δ).

### Formation of α_2_/δ puncta is independent of the GPI-anchoring motif

**Figure 3:**
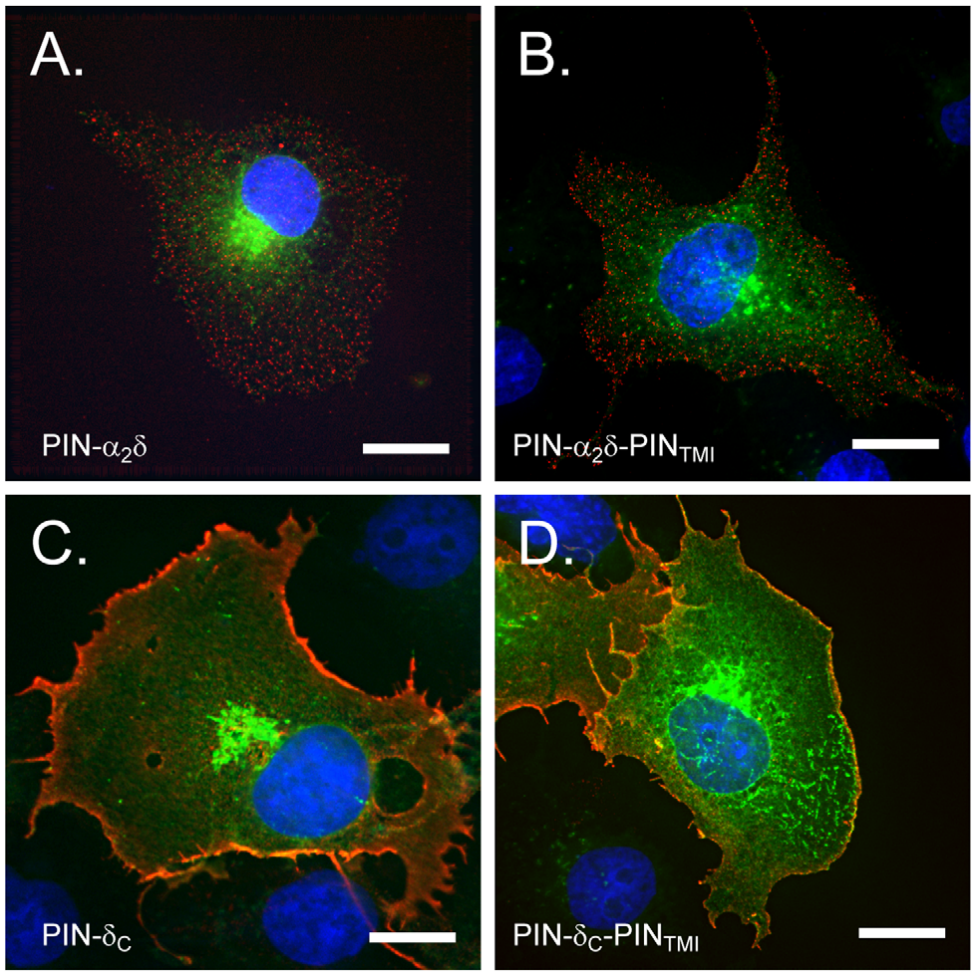
Surface and total cellular distribution of PIN-α_2_/δ chimera expressed in COS-7 cells. A. PIN-α_2_/δ. B. PIN-α_2_/δ-PIN_TMI_. C. PIN-δ_c_. D. PIN-δ_c_-PIN_TMI_. Cells were labelled with anti-HA and Cy5 secondary antibodies using a surface-labelling specific protocol (Methods) and the distribution of surface (red) and total (green, GFP) PIN construct expression determined by fluorescence imaging. Note strong labelling at cell margins for PIN-δ_c_ and PIN-δ_c_-PIN_TMI_ and highly punctate labelling for PIN-α_2_/δ and PIN-α_2_/δ-PIN_TMI_. Scale bar 15 µm.

Upon expression in COS-7 cells and surface anti-HA immunostaining, PIN-α_2_/δ exhibited a labelling pattern (Fig. 3A) characterised by the appearance of numerous small puncta, spread randomly over the cell surface, and matching that of WT α_2_/δ-1 [21]. In contrast, such puncta were absent in cells expressing PIN-δ_c_ (Fig. 3C) Rather, PIN-δ_c_ labelling was distributed evenly over the cell surface and at the cell margins. Significantly, the two different patterns of labelling seen between PIN-α_2_/δ and PIN-δ_c_ were retained in the derivative PIN-α_2_/δ-PIN_TMI_ (Fig. 3B) and PIN-δ_c_-PIN_TMI_ (Fig. 3D) constructs, where the GPI anchoring motifs had been disrupted.

### Raft localisation requires α_2_/δ sequences upstream of the GPI-anchoring motif

**Figure 4:**
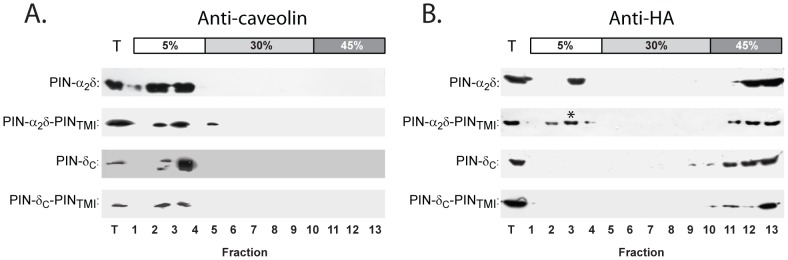
Distribution profile of PIN-α_2_/δ chimera in detergent-resistant membranes is not affected by disruption of the putative GPI anchoring motif. COS-7 cells were transfected with the corresponding PIN chimera and the membranes analysed via immunoblotting of fractions from sucrose density gradients containing 1% Triton-X-100, using antibodies to caveolin (endogenous) (Panel A) or anti-HA (Panel B)(for PIN chimera). Representative blots in panels A and B, correspond to cells transfected with PIN-α_2_/δ, PIN-α_2_/δ-PIN_TMI_, PIN-δ_c_ and PIN-δ_c_-PIN_TMI_. Note the absence of PIN-δ_c_ or PIN-δ_c-_PIN_TMI_ in raft fractions (3–6) and the presence in raft fractions of both PIN-α_2_/δ and PIN-α_2_/δ-PIN_TMI_ (asterisk in B). Immunodetection loading controls are denoted by ‘T’.

Elsewhere, we have shown an intimate link between the formation of puncta and the co-localisation of α_2_/δ with lipid raft proteins [21]. Consequently, the presence of puncta in constructs lacking the putative GPI anchoring motif (PIN-δ_c_-PIN_TMI_ and PIN-α_2_/δ-PIN_TMI_) and *vice versa* (PIN-α_2_/δ and PIN-δ_c_), prompted us to examine and compare their raft localisation more directly. To this end, we exploited the ability of lipid raft components, including α_2_/δ subunits [18], [20], [21], to migrate into low density fractions upon equilibrium centrifugation of cell lysates in sucrose density gradients containing ice-cold non-ionic detergents [14], notably Triton-X-100 [15], [56], [57]. Following centrifugation of lysates prepared at 48 h post-transfection, gradients were fractionated and fractions immunoblotted using anti-HA antibodies (Fig. 4). To control for gradient fidelity, each fraction was also analysed for the presence of the raft marker caveolin. Irrespective of the transfection condition, endogenous caveolin (22 kDa isoform) was detected as a single peak in fractions corresponding to the 5%–30% sucrose interface (Fig. 4A). In cells transfected with PIN-α_2_/δ (Fig. 4B blot i) approximately 20% of the anti-HA immunoreactivity was distributed at the 5–30% interface in caveolin-positive fractions, with the remainder locating to fractions of higher density centred on the 30–45% sucrose interface. In contrast, PIN-δ_c_ – which contains the putative GPI motif – was localised exclusively in the higher density non-raft fractions (Fig. 4B blot iii). Next we examined the distributions of constructs PIN-α_2_/δ-PIN_TMI_ (Fig. 4B blot ii) and PIN-δ_c_-PIN_TMI_ (Fig. 4B blot iv) - which lack the putative GPI-motif. In both cases raft/non-raft distributions of HA-immunoreactivity were the same as their parent constructs (PIN-α_2_/δ: raft + non-raft and PIN-δ_c_: non-raft, respectively). Thus, the raft localisation of PIN-α_2_/δ appears independent of the GPI motif. Conversely, the presence of the GPI motif in PIN-δ_c_ is insufficient to support raft localisation, implying that upstream sequences are required.

### The expression of PIN-α_2_/δ cell surface puncta is resistant to PI-PLC treatment

**Figure 5:**
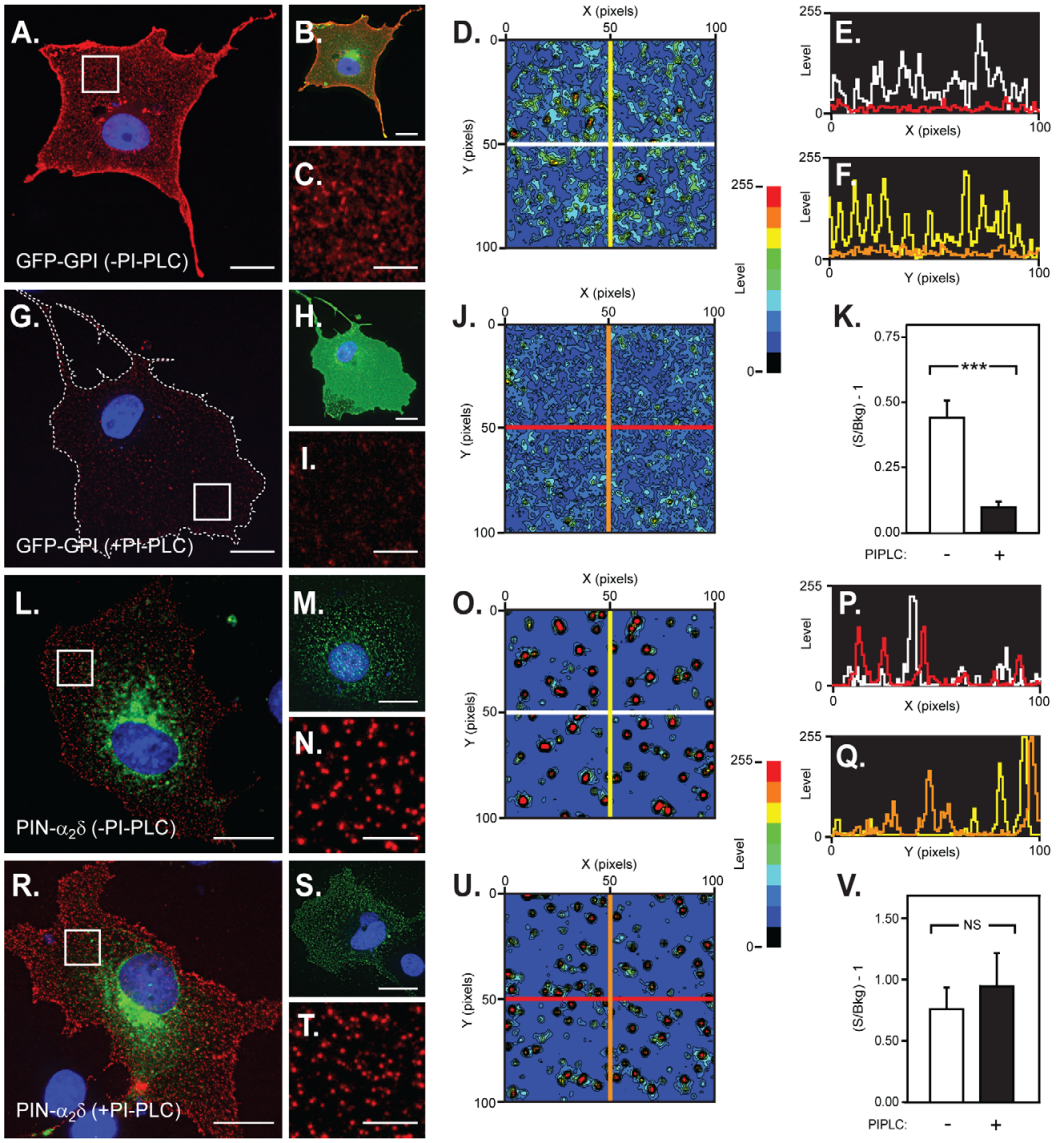
Effect of PI-PLC cell pre-treatment on the cell surface distribution of GFP-GPI (control) and PIN-α_2_/δ expressed in COS-7 cells. Panels A–I correspond to GFP-GPI fluorescence in the absence (A–C) and presence (G–I) of PI-PLC cell treatment. For clarity, panels A and G depict just the surface (red channel, anti-GFP) labelling corresponding to the merged (red (surface) and green (GFP, surface + intracellular) images shown in B and H. Panels C and I correspond to high magnification views of the boxed areas shown in A and G, respectively. Note strong surface labelling and evidence of clustering of GFP-GPI, in the absence of PI-PLC and diminution of surface cluster and interstitial fluorescence after PI-PLC treatment. Since contiguity between GFP-GPI clusters precluded standard particle analysis, the effect of PI-PLC on GFP-GPI clustering was analysed further by generating contour maps (panels D and J) (level scale (0–255) shown to right) of the labelling seen in panels C and I, respectively. Line scans based on the contour maps were then constructed to show differences in fluorescence intensity in the absence (white and yellow in D and F) or presence (red and orange in D and F) of PI-PLC cell treatment. Panel K shows the effect of PI-PLC cell pre-treatment on the signal to background fluorescence for raw images (n>8) collected using identical imaging conditions. *** denotes statistically significant difference (*P*<0.001); Student's t-test. Panels L–T correspond to images from cells transfected with PIN-α_2_/δ in the absence (L–N) and presence (R–T) of PI-PLC. Panels L and M (−PI-PLC) and R and S (+PI-PLC) show merged images for total (surface + intracellular)(green, GFP) and surface (red, anti-GFP)) for separate cells. Panels N and T correspond to high magnification views of the boxed areas shown in L and R (red, (surface) channel only). Note the presence of extensive PIN-α_2_/δ clustering irrespective of whether or not the cells had been treated with PI-PLC. Panels O and U correspond to contour maps (above) of the labelling seen in panels N and T, respectively (level scale (0–255) shown to right). Line scans corresponding to the contour maps were then constructed to show differences in fluorescence intensity in the absence (white and yellow in P and Q) or presence (red and orange in P and Q) of PI-PLC cell treatment. Panel V shows the effect of PI-PLC cell pre-treatment on the signal to background fluorescence for raw images (n>8) collected using identical imaging conditions. Note lack of effect of PI-PLC on PIN-α_2_/δ distribution (O and U) or intensity (V). All images are representative examples from data sets comprised of >8 images (>2 experiments). Scale bars are as follows: panels A, B, G, H, L, M, R and S, 20 µm; panels C, I, N and T, 4 µm.

Taken together, these data contradict the notion that the association of α_2_/δ-1 with lipid rafts is specified by the proposed GPI-anchoring motif [20]. To examine this issue further we tested for the existence of a GPI anchor through its susceptibility to PI-PLC cleavage [20], [58], [59]. First, we followed the approach of Davies et al., (2010) [20] who used imaging to assay the effect of PI-PLC on the surface expression of α_2_/δ constructs. For comparison we also examined the surface and total (surface + intracellular) distribution of GFP-GPI, a well-defined GPI-anchored green fluorescent protein [54]. As shown in Fig. 5A–D, GFP-GPI was found throughout the cell where it was localised in both tubulovesicular structures and at the cell surface. Although known to reside in lipid rafts like other GPI-anchored proteins [54], [59], [60], GFP-GPI surface labelling was not present in the well-defined puncta seen with PIN-α_2_/δ (e.g. Fig. 3), but rather it was distributed over the cell surface in a pattern reminiscent of a very fine, granular, meshwork (Fig. 5C,D). Following treatment with PI-PLC, all GFP-GPI-transfected cells showed a qualitative decrease in surface (Cy5/anti-GFP) labelling intensity and distribution compared with non-PI-PLC-treated cells (Fig. 5G–J). More quantitative comparisons based on determining the ‘on cell’ signal to noise (‘off cell’ background) ratio (S/B) of raw (i.e. non-background subtracted) images, showed that PI-PLC caused a reduction in GFP-GPI surface labelling intensity to 23% of control (i.e. −PI-PLC) levels ((S/B)−1 = 0.44±0.066 n = 8 (−PI-PLC) vs (S/B)−1 = 0.10±0.0217 n = 8 (+PI-PLC); p = 0.0002) (Fig. 5K). In parallel, we examined the action of PI-PLC on the surface expression of PIN-α_2_/δ. In contrast to GFP-GPI, and as noted above, PIN-α_2_/δ showed a pattern of surface labelling comprised of numerous high intensity puncta, with little interstitial (inter-punctal) labelling (Fig. 5L–O). Significantly, however, pre-treatment of cells with PI-PLC had no apparent effect on the labelling intensity ((S/B)−1 = 0.75±0.18 n = 6 (−PI-PLC) vs 0.95±0.27 n = 8 (+PI-PLC), p = 0.56) (Fig. 5 R–V and Fig. S2). Equally important, using detailed particle analysis we found no effect on the dimensions or density of the PIN-α_2_/δ puncta (Fig. S2). Neither the number of particles of given area (size distribution)(Fig. S2A), nor the particulate area fraction (a measure of changes in particle dimension) (Fig. S2B) were affected by PI-PLC treatment. Thus, we found no evidence for the effects predicted were PI-PLC treatment able to induce either ‘stripping’ (i.e. decreased particle size), disassembly (formation of smaller puncta) or both (Fig. S2C–F).

### The raft distribution of both PIN-α_2_/δ and caveolin in sucrose gradients is altered by PI-PLC treatment

**Figure 6:**
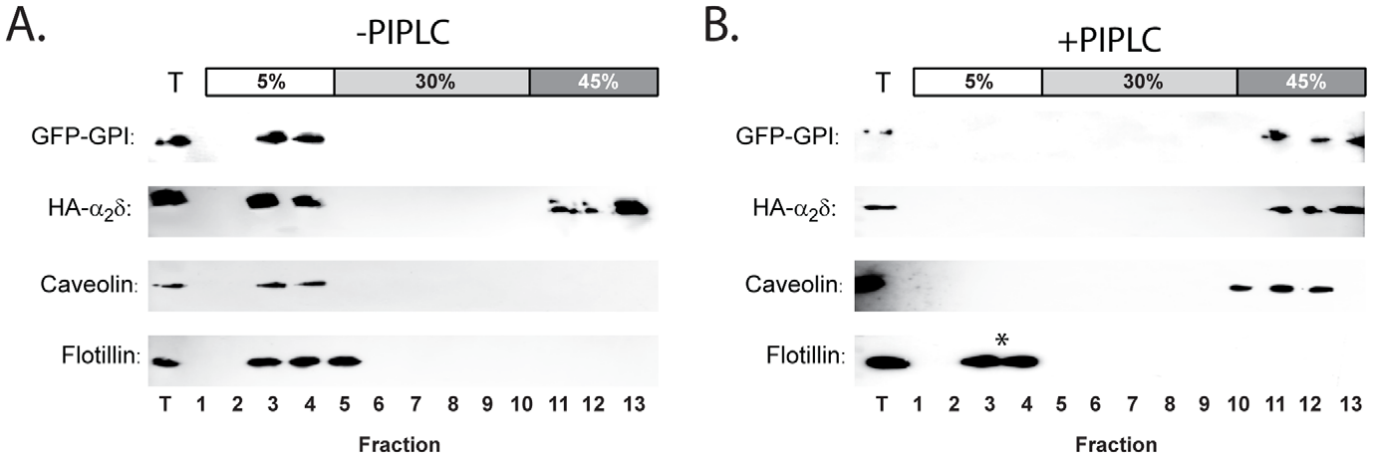
Effect of GPI-anchor removal through cell pre-treatment with PI-PLC. COS-7 cells were transfected with either GFP-GPI or HA-α_2_/δ and the membranes analysed via immunoblotting of fractions from sucrose density gradients containing 1% Triton-X-100, using antibodies to GFP (GFP-GPI), the HA-epitope tag (HA-α_2_/δ), caveolin (endogenous) or flotillin (endogenous). Representative blots at left and right correspond to cells before and after pre-treatment with PI-PLC, respectively. Note the presence of all proteins in the buoyant (raft) fraction prior to PI-PLC exposure and restriction of GFP-GPI, HA-α_2_/δ and caveolin, but not flotillin (B., asterisk) in denser non-raft fractions following PI-PLC exposure. Immunodetection loading controls are denoted by ‘T’.

As a further test for the presence of a GPI anchor in PIN-α_2_/δ, we examined the effect of PI-PLC on the partitioning of PIN-α_2_/δ in lipid raft fractions obtained using equilibrium centrifugation in sucrose gradients containing ice-cold Triton-X-100. As shown in Fig. 6, gradient analysis of lysates from cells expressing GFP-GPI (Fig. 6A, blot i) showed anti-GFP immunoreactivity exclusively in lipid raft fractions at the 5–30% sucrose interface. In contrast, lysates from cells pre-treated with PI-PLC (Fig. 6B, blot i) showed a marked shift in immunoreactivity which was now present in higher density non-raft fractions. Next, we examined lysates from cells transfected with PIN-α_2_/δ. As before (Fig. 4), anti-HA immunoreactivity was detected in both the raft and non-raft fractions ((Fig. 6A, blot ii). However, following pre-treatment of cells with PI-PLC all the anti-HA immunoreactivity appeared in the higher density, non-raft fractions (Fig. 6B, blot ii). While these data supported the contention that PIN-α_2_/δ is GPI-anchored [20], it was also possible that PI-PLC might have a more globally disruptive effect on lipid raft integrity, particularly given the lack of effect of molecular disruption of the GPI anchoring motif. To examine such a possibility we, therefore, examined the effect of PI-PLC on the gradient distribution of both caveolin (Fig. 6A,B, blot iii) and flotillin (Fig. 6A,B, blot iv) - two endogenous raft markers with separate and independent modes of raft association [61], [62], which both co-localise in puncta containing α_2_/δ [21]. As anticipated, both caveolin (Fig. 6A, blot iii) and flotillin (Fig. 6A, blot iv) were concentrated in raft fractions in the absence of PI-PLC pre-treatment. However, following PI-PLC pre-treatment, the distribution of caveolin (Fig. 6B, blot iii), but not flotillin (Fig. 6B, blot iv), shifted such that it was found primarily in the higher density non-raft fractions. Thus, PI-PLC appears to have a generally disruptive effect on the integrity of lipid rafts, whose detection depends upon whether caveolin or flotillin is used as a marker.

### Treatment with PI-PLC alters the cellular distribution of caveolin but not flotillin

**Figure 7:**
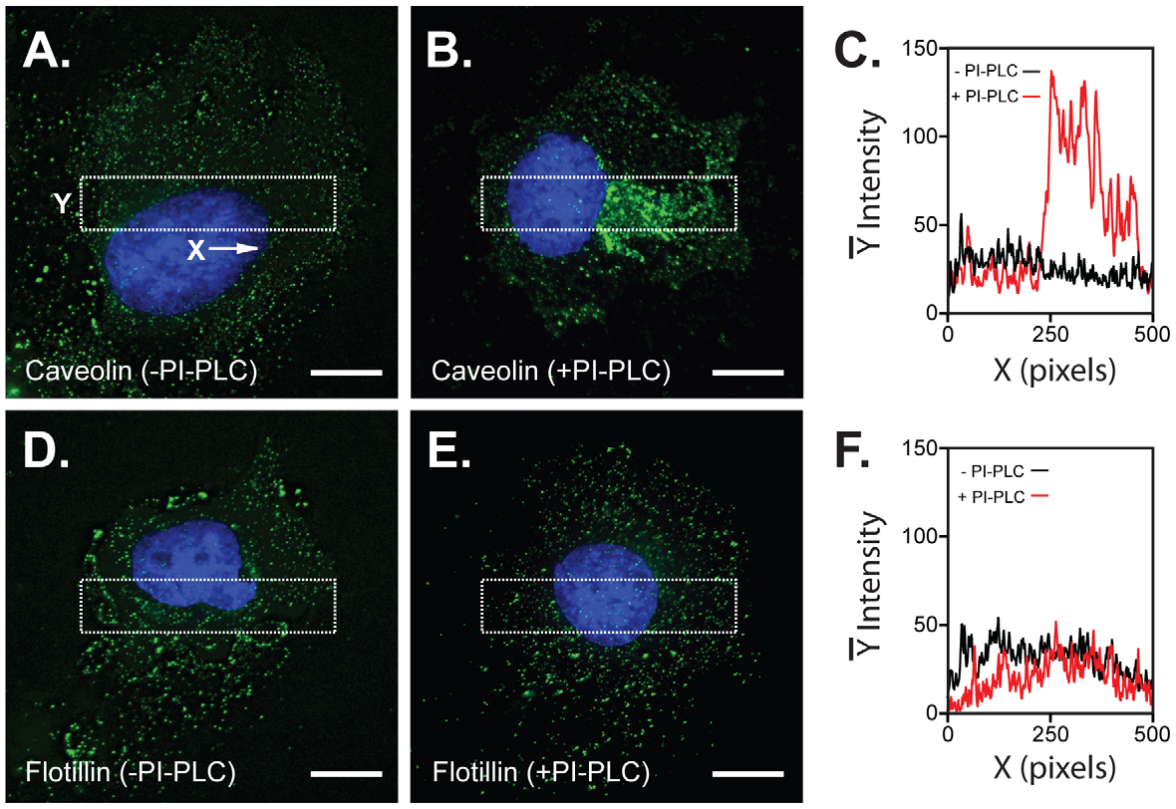
Effect of PI-PLC cell pre-treatment on the distribution of endogenous caveolin and flotillin in COS-7 cells. Panels A and B correspond to caveolin labelling in the absence (A) and presence (B) of PI-PLC cell pre-treatment. Panel C depicts intensity profiles (averaged in y axis) corresponding to boxes shown in A and B (red and black lines corresponding to profiles with and without PI-PLC, respectively). By averaging the fluorescence intensity, such ‘box scans’ reduce the noisiness seen in individual line scans. Note aggregation of caveolin fluorescence proximal to the nucleus (B) and increase in intensity (C) in images from cells pre-treated with PI-PLC. Panels D and E depict flotillin labelling in the absence (D) and presence (E) of PI-PLC cell pre-treatment. The corresponding box scans are shown in F (red line: +PI-PLC; black line: −PI-PLC). Note similarity in flotillin distribution irrespective of cell pre-treatment with PI-PLC. Scale bars: 15 µm.

To obtain further evidence for a generalised effect of PI-PLC on raft integrity, we examined the cellular distribution of caveolin and flotillin before and after PI-PLC treatment, using imaging assays (Fig. 7). As documented elsewhere [21], both of these raft marker proteins localise to puncta and large aggregates throughout permeabilised, non-PI-PLC-treated, COS-7 cells (Fig. 7A (caveolin), 7D (flotillin)). However, following pre-treatment of cells with PI-PLC there was a marked alteration in caveolin labelling to patterns consisting of patches of intense labelling proximal to the cell nucleus and the appearance of more diffuse labelling over the cell surface (Fig. 7B). In contrast, pre-treatment of cells with PI-PLC had no effect on the distribution of flotillin (Fig. 7E) which remained punctate throughout. These data are therefore consistent with those from the sucrose-density gradient experiments and support the notion that PI-PLC – a primary tool for defining α_2_/δ-1,2 and 3 as GPI-anchored proteins [20] - has indirect effects which may confound the assignment of proteins as possessing GPI anchors.

## Discussion

In this study we have tested the notion that the Ca_v_α_2_/δ-1 subunit is a GPI anchored protein, by substitution of the putative GPI-anchoring motif, including the downstream sequence formerly designated as TM-spanning, with a *bona fide* [51] TM-spanning and intracellular sequence from the trafficking reporter, PIN-G. Using fundamentally different algorithms, each chimera is predicted to have little or no GPI anchoring potential, due to direct disruption of all residues adjacent and subsequent to the putative ω+1 site and the extended intracellular domain. By replacing the GPI-anchoring motif with bulky lysine and hydrophobic amino acids throughout, our chimera, therefore, represent an even more extensive alteration of both the motif structure and the overall GPI anchoring potential (Table 1) than that achieved previously in Ca_v_α_2_/δ-2 and Ca_v_ α_2_/δ-3, where just three ω site residues were mutated [20]. Furthermore, by generating PIN chimera corresponding to a full length or truncated α_2_/δ subunit, both containing the putative GPI-motif, it was possible to examine the independence of this motif from upstream residues.

Significantly, PIN-α_2_/δ supports the key hallmarks of WT α_2_/δ-1 functionality, notably a 4-fold enhancement of peak current density, a hyperpolarising shift in *V*_50_ for activation and an enhanced rate of inactivation, when co-expressed with Ca_v_2.2/β_1b_ subunits. Such current enhancement arises through direct actions on anterograde and retrograde trafficking of Ca_v_ complexes [6], [63] and is highly susceptible to post-translational modification events [55]. Thus, PIN-α_2_/δ is evidently able to undergo processing and trafficking events similar to WT α_2_/δ-1 and like WT α_2_/δ-1, can co-assemble with Ca_v_2.2 α_1_ subunits. Equally significant, the Ca_v_2.2/β_1b_ current enhancement and kinetic features imparted by the GPI-anchor-deficient PIN-α_2_/δ-PIN_TMI_ construct are identical to those of PIN-α_2_/δ. This is especially remarkable given that the association of α_2_/δ-1 with lipid rafts has been directly attributed to GPI-anchoring [20] and that disruption of either rafts [16], [18]–[21], or GPI-anchoring [20], has been reported to affect Ca_v_ current density. Our observation that PIN-δ does not support enhancement of Ca_v_2.2/β_1b_ currents is entirely consistent with the known requirement for sequences in the α_2_ subunit [7], [28], [29].

In both our biochemical and imaging assays PIN-α_2_/δ exhibits the raft-association characteristics of WT α_2_/δ-1 [21]. However, in these assays PIN-δ_c_ - which contains the putative GPI-anchoring motif and 46 (33 δ_c_ and 13 GFP-linker) upstream residues between GFP and the predicted ω site (CGG) showed no raft localisation. In contrast, GFP-GPI, which contains just 22 residues between GFP and the ω site, is raft localised. Thus, raft localisation must depend upon additional determinants upstream of the δ_c_ sequence rather than merely the number of residues upstream of the ω site. Although it is conceivable that determinants upstream of δ_c_ somehow promote GPI-anchor attachment, our observation that raft localisation is conserved in both PIN- α_2_/δ and the anchor-deficient PIN-α_2_/δ-PIN_TMI_, argues strongly against any involvement of the putative GPI-anchor motif reported by Davies et al (2010) [20]. While we cannot rule out the possibility of cryptic (i.e. internal) GPI-anchor motifs these are very rare and are thought to resemble the classic carboxy terminal anchoring motifs in structure [64], [65]. Indeed, using predictive algorithms to assess the GPI-modification potential for sequentially truncated α_2_/δ-1 constructs, we have been unable to detect any additional regions within δ-1 that could serve as obvious GPI-anchoring motifs (Fig. S3).

Notwithstanding the above, our data do not exclude the possibility that GPI-anchoring plays an indirect role in α_2_/δ raft localisation. Indeed, upon treatment with PI-PLC, PIN- α_2_/δ was no longer associated with lipid rafts when assessed by sucrose gradient analysis. While this effect has been interpreted as arising via the release of α_2_ and regions of δ-1 up to the ω site [20] (Table 2), it appears to be non-specific since PI-PLC also prevented the raft-association of caveolin which, in contrast to GPI-anchored proteins, is localised to the inner membrane leaflet [61]. Significantly, depletion of caveolin has been reported to re-distribute Type I TM proteins from raft to non-raft fractions [66] which may explain the data reported by Davies et al., [20] where flotillin was the primary raft marker (Table 2). In support of this, our images showing that PI-PLC causes partial dispersal of caveolin, are highly reminiscent of those obtained from COS-7 cells treated with the cholesterol-depleting agent, methyl-β-cyclodextrin (M-β-CD) [21]. However, while M-β-CD also disperses flotillin and prevents its co-localisation in lipid raft fractions, PI-PLC does not. Thus, PI-PLC treatment can disrupt raft integrity, but not completely. To our knowledge, potentially disruptive effects of PI-PLC on raft structure have not been examined, although phospholipase C activity and low concentrations of its end product - diacylglycerol, are known to destabilise model membranes including those containing raft lipids [67]. Quite why caveolin and flotillin should show differential raft partitioning after PI-PLC treatment is also unclear, but likely reflects their differing modes of membrane association. While both proteins are acylated, only caveolin has a transmembrane domain [61], [62], [68]. Irrespective of the mechanisms, a differential effect of PI-PLC on caveolin and flotillin raft localisation, clearly, warrants caution when using these markers alone to assess raft integrity.

**Table 2:** Comparison of experimental approaches and conclusions in the present study and that of Davies et al., [20].

	This study	Davies et al. [20]
**A) Substrates**		
Constructs	HA α_2_/δ-1 (Rat), PIN α_2_/δ-1 chimera	No constructs or mutants employed(point mutants in just α_2_/δ-2/3)
Cell types used	COS-7	Rat DRG, Hippocampus, tsA-201 cells, cardiac muscle (data not shown)
Immunodetection	Anti-HA/Anti-GFP	Anti- α_2_/δ-1
**B) Evidence for GPI-Anchoring motif in** α_2_/δ**-1**		
Algorithms	Probable in only 1/3 algorithms	Not given
Other	Not inferred (see Introduction)	Inferred from α_2_/δ-2, α_2_/δ-3 data and partial homology.
**C) Raft isolation**		
Cells	COS-7 cell lysates	Hippocampal tissue lysates, tsA-201 cell lysates, cardiac muscle (data not shown)
Detergent	Triton-X-100, 4°C	Triton-X-100, 4°C
Raft markers	Endogenous Caveolin and Flotillin-1	Endogenous Flotillin-1
Conclusions	Localisation of α_2_/δ-1 in raftsRaft localisation independent of GPI anchoring motif	Localisation of WT α_2_/δ-1 in rafts
**D) Imaging**		
Cells	COS-7	Rat DRG
α_2_/δ-1	Transfected constructs	Endogenous
Labelling method	Surface protocol	Non-permeabilised*
Detection	Immunofluorescence	Immunofluorescence
Quantification	Intensity and Particle analysis	Intensity
Conclusions	Formation of α_2_/δ-1 puncta independent of GPI anchoring motif but requires upstream sequences	N/A
**E) PI-PLC**		
Concentration	4 U/ml, 1 h, 37°C	4–8 U/ml, 1 h, 37°C
Treatment - rafts	Live COS-7 cells prior to lysis	Hippocampal tissue lysates
Conclusions	Raft localisation of α_2_/δ-1 reduced	Raft localisation of α_2_/δ-1 reduced
Treatment - imaging	COS-7 cells, surface protocol	‘non-permeabilised’* DRG cells
Conclusions	Formation of surface α_2_/δ-1 puncta resistant to PI-PLC	Surface expression of α_2_/δ-1 reduced by PI-PLC
**F) Electrophysiology**		
Constructs	Ca_v_2.2/β_1b_ +/− PIN- α_2_/δ-1 chimera, or, WT-α_2_/δ-1	α_2_/δ-1 not tested.
Cells	COS-7	tsA-201
Conclusions	Current density unaffected by loss of GPI anchoring motif	Not tested (reduced current density in α_2_/δ-2/-3 on disruption of GPI anchoring motif)

Key differences are our use of: a) both caveolin and flotillin as raft markers, b) a carefully controlled surface-labelling protocol, c) lysates from live cells treated with PI-PLC and d) the extensive use of chimera which ablate the purported GPI-anchoring motif. Asterisks denote the use of non-permeabilised cells without reference to controls. As we show elsewhere [21], fixative alone can cause significant cell permeabilisation.

Taken together, our chimera studies show that Ca_v_ α_2_/δ-1 raft localisation is independent of the putative GPI-anchoring motif and that this motif does not localise chimera to rafts. By inference, our data do not support the revised model for the topology, membrane association (i.e. GPI anchoring) or ability of α_2_/δ-1 subunits to target Ca_v_s to lipid rafts. Rather, raft association – at least for α_2_/δ-1 - appears to require sequences upstream of the ω site that most likely mediate protein-protein rather than lipid-lipid interactions, a scenario more consistent with emerging views of raft biogenesis and aggregation [14], [42], [69].

## Materials and Methods

### Chemicals

The construct encoding wild-type rat Cav α_2_/δ-1 (Neuronal splice variant; Genbank accession number: NM_012919.2) in pcDNA3.1 was supplied by T.P. Snutch (Univ. British Columbia, Canada). Rabbit Ca_V_2.2 in pMT2 (D14157), rat Cavβ_1b_ in pMT2 (X61394) and the mut-3 variant of GFP-pMT2 (U73901) were supplied by A.C. Dolphin (University College London, UK). The pcDNA3.1 plasmid was obtained from Invitrogen, UK. Primary antibodies were obtained from the following sources: anti-α_2_/δ-1 (Upstate/Millipore, UK), anti-flotillin-1, anti-clathrin, anti-GFP (Sigma-Aldrich, UK) and anti-HA (Covance, UK). Secondary antibodies were obtained as follows: FITC-conjugated anti-rabbit and anti-mouse IgGs (Jackson Immunoresearch, UK), Cy5-conjugated anti-mouse and anti-rabbit IgG (Jackson Immunoresearch, UK) and horseradish peroxidase (HRP)-conjugated anti-rabbit and anti-mouse IgGs (Dako, UK). All other reagents were obtained from Sigma-Aldrich, UK, unless stated otherwise.

### Molecular biology

An α_2_/δ-1 construct bearing an HA epitope tag between amino acid residues I612 and K613, was generated using a three step strategy as described in Robinson et al. (2010) [21]. All PIN constructs were prepared through the sequential insertion, deletion or substitution [70] of specified rat α_2_/δ-1 sequences into the PIN-G plasmid (Genbank: AY841887), using the QuikChange™ II kit (Agilent Technologies, UK) and mutagenic megaprimers prepared by PCR. Construct fidelity was confirmed by in-house sequencing (see Fig. 1 and Fig. S3C for chimera junctions).

### Cell culture and transient transfection

Culture and transient transfection of COS-7 cells (European Cell Culture Collection, Health Protection Agency, U.K.), were carried out as described in Robinson et al. (2010) [21]. Transient transfections were performed in serum-free Dulbecco's modified Eagle's medium (DMEM) at a cell confluency of 60–70% using FuGene 6 (Roche Diagnostics, U.K.; imaging and electrophysiology) or Turbofect (Fermentas, U.K.; biochemical experiments) at a total DNA∶reagent ratio of 1∶3 (w/v), (total DNA: 2 µg for 6-well plates/35 mm dishes, 12 µg DNA for 10 cm plates). Transfections with Ca_v_2.2, Ca_v_β_1b_ and Ca_v_α_2_/δ-1 used a ratio of 3∶1∶1 by mass of subunit cDNA. For transfections omitting α_2_/δ cDNA, the α_2_/δ cDNA was replaced with pcDNA3.1 to maintain the equivalent mass ratio. Cells were maintained at 37°C, 5% CO_2_ in complete medium for a total of 48 hours (including any re-plating step), after which cells were: a) fixed for microscopy (below), b) re-plated onto 22 mm square coverslips for electrophysiology, or c) lysed for biochemical experiments. For re-plating post-transfection, cells were detached using a non-enzymatic cell dissociation solution (Sigma Aldrich, UK) before re-seeding in fresh complete medium.

### Western immunoblotting

At 48 h post-transfection, COS-7 cells were washed in PBS and lysed at 4°C in a radio-immunoprecipitation assay (RIPA) buffer with Complete MINI EDTA-free protease inhibitor cocktail (Roche, UK). The cell lysates were then passed through a 22-gauge syringe needle 10 times to shear genomic DNA, and centrifuged at 1000 g_av_. Supernatants were then incubated at 37°C for 15 min with Laemmli loading buffer containing 20 mM DTT and then heated to 95°C for 2 min. Sample proteins were resolved by SDS-PAGE on 10% Tris-HCl gels for 80 min at 160 V (Mini-Protean cell, BioRad, UK) and then transferred by electrophoresis (100 V for 2 h) onto nitrocellulose membranes (Whatman, UK). Air dried membranes were immersed overnight in blocking buffer (5% non-fat dry milk in Tris-buffered saline (TBS) with 0.1% Tween-20 (TTBS)), washed three times with TTBS and then incubated with the appropriate primary antibody in TTBS for 1 h at 20°C. The membranes were then re-washed with TTBS and incubated for 1 h at 20°C with the appropriate secondary HRP-conjugated antibody (1∶1000) in TTBS. After further washing with TTBS, the membranes were treated with Western Lightning enhanced chemiluminescence reagent (Perkin Elmer, UK) and immunoreactive proteins detected by exposure to film (GE Life Sciences, UK).

### Sucrose gradient fractionation

As we described recently [21], transiently transfected COS-7 cells were washed in PBS and lysed 48 h post-transfection with MBS (Mes-buffered saline: 25 mM Mes, pH 6.5, 150 mM NaCl) with 1% Triton-X-100 at 4°C. For a single experiment, 9×10 cm dishes were used and 150 µl of MBS/Triton-X-100 was added to lyse the cells. Cells were scraped off the dish, passed through a 22-gauge needle 10 times to shear genomic DNA and 450 µl of lysate was reserved for use as a control. The remaining 900 µl of lysate was mixed with 900 µl of 90% sucrose/MBS (w/v), placed in a 5 ml polypropylene centrifuge tube (Sorvall) and carefully overlaid with 1.5 ml of 30% sucrose/MBS, followed by 1.5 ml of 5% sucrose/MBS. Gradients were spun at 38,500 rpm (140,000 g_av_) in a Sorvall Discovery 100SE ultracentrifuge using an AH-650 rotor for 16 h at 4°C. Post-centrifugation, 15 fractions were taken from top to bottom of the tube and analysed in subsequent Western immunoblotting. To concentrate proteins, fractions were incubated with 25% trichloroacetic acid (final), at 4°C for 30 min. Samples were centrifuged at 14,000 rpm (13,000 g_av_) at 4°C for 20 min and the pellets washed twice with ice-cold acetone, ensuring not to disrupt the pellets. Pellets were dried at 42°C for 10 min before re-suspension in 50 µl of MBS and analysed by Western immunoblotting.

### Immunocytochemistry

Cells for fluorescence microscopy were re-plated 24 hours post-transfection onto 13 mm coverslips coated with 0.01% poly-L-lysine. To preclude fixation artefacts, all imaging experiments of surface expression were performed using a two-step protocol [21]. Briefly, COS-7 cells (48 h post-transfection) were cooled on ice to 4°C and after 10 min, treated with primary antibody diluted in PBS. After 1 h at 4°C, coverslips were washed 3 times with PBS and the cells fixed with 4% (w/v) paraformaldehyde for 20 min at 20°C. Cells were then treated with the appropriate (Cy5 or FITC) fluorophore-conjugated secondary antibody for 1 h at 20°C. In order to detect intracellular epitope expression, cells were permeabilised post-fixation with 0.5% saponin for 10 min at 20°C, prior to incubation with primary antibody. Nuclear staining was performed with DAPI (4′,6-diamidino-2-phenylindole; 1 µg/ml) for 2 min at 20°C, prior to mounting with Prolong Gold Antifade reagent (Invitrogen/Molecular Probes).

### PI-PLC treatment

At 48 h post-transfection, COS-7 cells were washed with serum-free DMEM and incubated with PI-PLC (Invitrogen, U.K.; 4 Units/mL) for 1 h at 37°C. The cells were then washed in DMEM to remove PI-PLC, placed on ice and processed for imaging (above) or immunoblotting.

### Fluorescence deconvolution microscopy and image analysis

Images of cells on coverslips were acquired on a Delta Vision RT (Applied Precision, Image Solutions, UK) restoration microscope using a ×60 objective lens and appropriate wavelength filters. The images were collected using a Coolsnap HQ (Photometrics) camera with a Z optical spacing of 0.1 µm. Raw images were then deconvolved using Softworx software and displayed as maximum projections using NIH Image J ((W.S. Rasband, NIH Bethesda, USA; Wright Cell Imaging facility bundle: http://www.uhnres.utoronto.ca/facilities/wcif.htm).

### Whole-cell patch-clamp electrophysiology

As described previously [21], COS-7 cells were transiently transfected with Ca_v_2.2∶β_1b_∶ α_2_/δ-1∶mut3-GFP-pMT2 cDNA in a 3∶1∶1∶0.2 mass ratio and current recordings made 48 h post-transfection. Where α_2_δ-1 or mut-3 GFP was omitted, empty pcDNA3.1 vector was substituted to maintain the equivalent mass of DNA. Electrophysiological recordings of barium currents were made from green fluorescent COS-7 cells, using the whole-cell configuration of the patch clamp technique and the following solutions [71]. The internal solution contained (mM): caesium aspartate 140.0; EGTA 5.0; MgCl_2_ 2.0; CaCl_2_ 0.1; Hepes 20.0; K_2_ATP 1.0; adjusted to pH 7.2 with CsOH and 310 mosm.l^−1^ with sucrose. The external solution contained (mM): TEABr 160.0; MgCl_2_ 1.0; KCl 5.0; NaHCO_3_ 1.0; Hepes 10.0; glucose 4.0; BaCl_2_ 10; adjusted to pH 7.4 with Tris-base and to 320 mosm l^−1^ with sucrose. All experiments were performed at room temperature (20–22°C). An Axopatch 200B amplifier (Molecular Devices, Palo Alto, CA, USA) was used for recordings which were filtered at 2 Hz and digitised at 2–44 kHz using a Digidata 1440A A/D converter (Molecular Devices). Standard current-voltage protocols involved 150 ms sweeps from a holding potential, *V*_h_ of −80 mV to command voltages of −30 to +65 mV in 5 mV steps. Current density-voltage (*I-V*) relationships for each cell were fitted with a Boltzmann function:
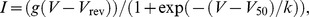
Where, *V*_rev_ is the reversal potential, *V*_50_ is the voltage for half maximal activation of current, *g* is the conductance, and *k* is the slope factor.

Data acquisition and analysis was performed using pCLAMP software (version 10, Molecular Devices) and Origin (version 7.0, Microcal, Northampton, MA, USA).

### Data analysis

All data are presented as the mean ± standard error of the mean (S.E.M) for *n* trials. Statistical analysis was carried out by Student's t-test or ANOVA (one-way with Student-Newman-Keuls (SNK) *post hoc* correction), as appropriate, using 95% confidence limits (SigmaStat software, Jandel Scientific). Contour mapping was performed using Origin V.8 (OriginLab Corp., MA) on images converted from TIFF format to 2D matrices using the TIFFDump algorithm written by J.S Wadia [72]. Particle analysis was performed on thresholded images using NIH Image J.

## Supporting Information

Figure S1 **Effect of the GFP-tag on the biophysical properties of Ca_v_2.2/β_1b_ channels co-expressed with PIN-α_2_δ and PIN-α_2_δ-PIN_TMI_.** *(A)* Average current density-voltage (*I-V*) plots for Ca_v_2.2/β_1b_ currents co-expressed with PIN-α_2_δ (open circle) versus PIN(deGFP)-α_2_δ (closed circle). *(B)* Average I-V plots for Ca_v_2.2/β_1b_ currents co-expressed with PIN-α_2_δ-PIN_TMI_ (open circle) versus PIN(deGFP)-α_2_δ-PIN_TMI_ (closed circle). Continuous lines indicate Boltzmann fits to *I-V* plot using the function described in the Methods. Panels *C*, *D*, show representative peak current traces for PIN-α_2_δ versus PIN(deGFP)-α_2_δ (red) and PIN-α_2_δ-PIN_TMI_ versus PIN(deGFP)-α_2_δ-PIN_TMI_ (red), respectively. Histograms of the time constants of activation (τ_act_) and inactivation (τ_inact_) at peak current density for PIN(deGFP)-α_2_δ-deGFP versus PIN-α_2_δ *(E)* and PIN(deGFP)-α_2_δ-PIN_TMI_ versus PIN-α_2_δ-PIN_TMI_-deGFP *(F)*, where GFP-tagged (cross-hatched) and deGFP (red). τ_act_ and τ_inact_ were fitted with a single exponential function. Asterisks denote statistically significant differences (Student's t-test; ** = *P*<0.01; *** = *P*<0.001). Currents were evoked using 150 ms depolarising steps in 5 mV intervals (−30 to +65 mV), from a holding potential, *V*_h_, −80 mV. Data are shown as the mean ± S.E.M.
(TIF)

Figure S2
**Particle analysis of PIN-α_2_δ cell surface clustering in the presence and absence of PI-PLC cell pre-treatment.** **A.** Effect of PI-PLC on the size distribution of PIN-α_2_δ particles. Inset: data re-plotted using log scale. To facilitate overlay of images from separate cells, the number of particles Np_i_, of given area (Ap_i_, (abscissa) in pixel^2^) is expressed as a percentage of the total (N_t_ where N_t_ = Σ N_Ai_). Note overlap in data, irrespective of pre-treatment with PI-PLC. **B.** Distribution of fractional coverage represented by PIN-α_2_δ particles. Inset: data re-plotted using expanded scale. Here and elsewhere [21], we define fractional coverage as the % of the total particulate area (C_t_) within a region of interest (ROI), (not the area of the ROI) accounted for by particles of area Ap_i_ (i.e. Np_i_.Ap_i_/C_t_, where 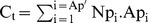 and Ap' is the area of the largest particle in the data set). Using this representation it is possible to discriminate cases where coverage of the total particle area arises from many small particles or a lesser number of larger particles. For example, in the simple situation where there are 4 particles each of size 10 pixel^2^ and 1 particle of size 60 pixel^2^, then C_t_ = 100, then for the smaller particles Np_i_/N_t_ = 0.8 and the fractional coverage = 0.4, for the larger particle Np_i_/N_t_ = 0.2 and fractional coverage = 0.6. In contrast, if the same total particulate area is comprised of 60 particles each of size 1 pixel^2^ and 4 particles each of size 10 pixel^2^, then Np_i_/N_t_ = 0.94 and the fractional coverage = 0.6, for the larger particles Np_i_/N_t_ = 0.06 and fractional coverage = 0.4). Note overlap of data, irrespective of pre-treatment with PI-PLC. Particle analysis was performed with Image J, using the adaptive thresholding plug-in, with thresholded images checked visually for accuracy. All data were extracted from 3 images from separate experiments. **C.** and **D.** Computer modelling of the effects of particle re-distribution on fractional coverage Fractional coverage graphs (D) were determined for the three particle size distributions shown in C. Note marked, and well-defined effect of particle re-distribution. For simplicity, the distribution curves in C were generated using equations based on a binomial distribution with terms p^4^ (black), 6p^2^q^2^ (red) and q^4^ (blue), (where q = 1-p), respectively. In each case, the number of particles N_t_ was adjusted to give an identical total particulate area, C_t_ = 1×10^5 ^pixel^2^ ((N_t_ = pixel^2^, black, red and blue curves, respectively). **E.** and **F.** Computer modelling of the effects of a reduction in particle area. In these simulations the number of particles was held constant (Nt = 10^4^), but the area of each decreased by 50% to mimic a ‘stripping’ effect such as that which might be seen with PI-PLC. Curves in E. were generated as in C., for the p^4^ binomial distribution. From comparisons of the size distribution and fractional coverage determined experimentally (A, B) and predicted from simulations (C–F), there is no evidence that PI-PLC pre-treatment has any effect on PIN-α_2_δ particle properties.(TIF)

Figure S3 **Comparative analysis of GPI-anchoring motifs.** A. Comparison of carboxy-terminal sequences of known GPI-anchored proteins and rat α_2_δ-1,2,3 & 4 showing the ω site(s) (red lettering) and hydrophobic regions (grey boxes) of the GPI-anchoring motifs. All dataset examples (i.e. non-α_2_δ) correspond to proteins where the ω site has been verified, experimentally. (References: P21589: Misumi, Y. et al. (1990) Eur. J. Biochem. 191:563–569; P22748: Okuyama, T. et al. (1995) Arch. Biochem. Biophys. 320:315–322; P08174: Moran, P. et al. (1991) J. Biol. Chem. 266:1250–1257; P04058: Mehlert, A., et al. (1993) Biochem. J. 296:473–479; P15328 & P14207: Yan, W. and Ratnam, M. (1995) Biochemistry 34:14594–14600; P01831: Williams, A.F. and Gagnon, J. (1982) Science 216:696–703; P16444: Adachi, H. et al. (1990) J. Biol. Chem. 265:15341–15345; P31358: Xia, M.Q. et al. (1993) Biochem. J. 293:633–640; P04273: Stahl, N. et al. (1990) Biochemistry 29:8879–8884; P13987: Sugita, Y. et al. (1993) J. Biochem. 114:473–477; P05187: Micanovic, R. et al. (1990) Proc. Natl. Acad. Sci. U.S.A. 87:157–161; P14384: Tan, F. et al. (2003) Biochem. J. 370:567–578; XP_001352170.1: Hall, N. et al. (2002) Nature 419:527–531). The α_2_δ-1-3 ω sites have been tested, experimentally (Davies et al., 2010, Robinson et al., 2010 above), while that for α_2_δ-4 is inferred based on sequence homology to α_2_δ-3. B. Left panel: Potential for GPI-modification for dataset and α_2_δ proteins shown in A inferred using Big-Pi predictor software (http://expasy.org/tools/). Proteins with positive or negative GPI modification potential are shown in blue and red, respectively. Asterisks denote proteins where the ω site differs from that inferred. Right panel detailed sequence comparison of inferred (red lettering) and predicted (asterisks) ω sites. In most cases the inferred ω site is very close (<2 residues) to that found experimentally. C. Analysis of potential upstream GPI-anchoring motifs in the delta subunit of WT α_2_δ-1 (or PIN-α_2_δ)(blue) and PIN-α_2_δ-PIN_TMI_ (red). Here, the GPI anchoring potential was determined (using Big-Pi [29]) as a function of successive truncation (1 residue at a time) of the carboxy terminus. Note: based on the length of the GPI-anchoring motif, any ω site is predicted to lie 20–30 residues upstream of the position of the indicated carboxy-terminal residue (abscissa). For simplification, the carboxy-terminal sequences have been re-numbered starting at residue 922 in WT α_2_δ-1 as shown in the corresponding sequences (i. and ii. right panel). For PIN-α_2_δ, the reported GPI-anchoring motif is shown in blue lettering. For PIN-α_2_δ-PIN_TMI_ green lettering denotes residues derived from PIN-G. In i. and ii., the grey boxes denote hydrophobic regions. With the exception of sequences near the junction of the α_2_ and δ delta subunits, all regions have a much lower GPI-modification potential than the WT α_2_δ C-terminus suggesting the likely absence of additional upstream GPI-anchoring motifs unmasked by proteolytic cleavage. Note, the low GPI-modification potential of both the non-truncated PIN-α_2_δ and PIN-α_2_δ-PIN_TMI_.
(TIF)

Table S1Biophysical properties of Ca_v_2.2/β_1b_ channels co-expressed with WT α_2_δ-1, PIN-α_2_δ, PIN-α_2_δ-PIN_TMI_ and PIN-δ. *I*_max_ is the maximum peak current density. Individual current density-voltage plots were fitted with a Boltzmann function:
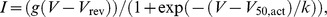
where *V*_rev_ is the reversal potential, *V*_50,act_ is the voltage for half maximal activation of current, *g* is the conductance, and *k* is the slope factor. Statistical analysis used Students unpaired t-test. Asterisks denote statistically significant differences from (−)α_2_δ-1, as follows: * = *P*<0.05, *** = *P*<0.001. *n* is the number of cells tested per treatment.(DOC)
