# Supplementary figures and images for: Targeting of Voltage-Gated Calcium Channel α2δ-1 Subunit to Lipid Rafts Is Independent from a GPI-Anchoring Motif
Source: PLoS One. 2011 Jun 10;6(6):e19802. doi: 10.1371/journal.pone.0019802 (PMC3112168; doi:10.1371/journal.pone.0019802)

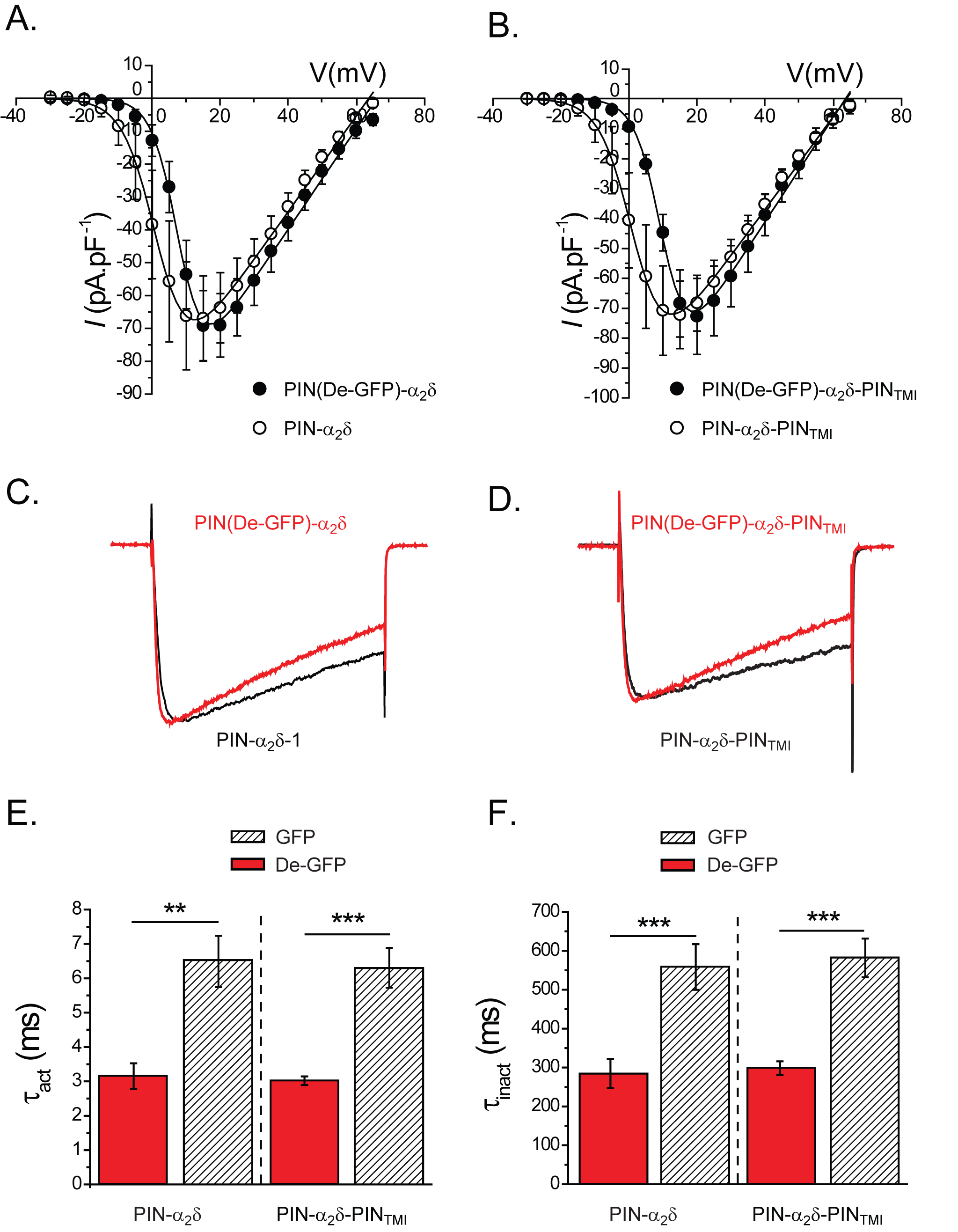

Supplement: Figure S1 — Effect of the GFP-tag on the biophysical properties of Cav2.2/β1b channels co-expressed with PIN-α2δ and PIN-α2δ-PINTMI. (A) Average current density-voltage (I-V) plots for Cav2.2/β1b currents co-expressed with PIN-α2δ (open circle) versus PIN(deGFP)-α2δ (closed circle). (B) Average I-V plots for Cav2.2/β1b currents co-expressed with PIN-α2δ-PINTMI (open circle) versus PIN(deGFP)-α2δ-PINTMI (closed circle). Continuous lines indicate Boltzmann fits to I-V plot using the function described in the Methods. Panels C, D, show representative peak current traces for PIN-α2δ versus PIN(deGFP)-α2δ (red) and PIN-α2δ-PINTMI versus PIN(deGFP)-α2δ-PINTMI (red), respectively. Histograms of the time constants of activation (τact) and inactivation (τinact) at peak current density for PIN(deGFP)-α2δ-deGFP versus PIN-α2δ (E) and PIN(deGFP)-α2δ-PINTMI versus PIN-α2δ-PINTMI-deGFP (F), where GFP-tagged (cross-hatched) and deGFP (red). τact and τinact were fitted with a single exponential function. Asterisks denote statistically significant differences (Student's t-test; ** = P<0.01; *** = P<0.001). Currents were evoked using 150 ms depolarising steps in 5 mV intervals (−30 to +65 mV), from a holding potential, V h, −80 mV. Data are shown as the mean ± S.E.M. (TIF) [file pone.0019802.s001.tif]

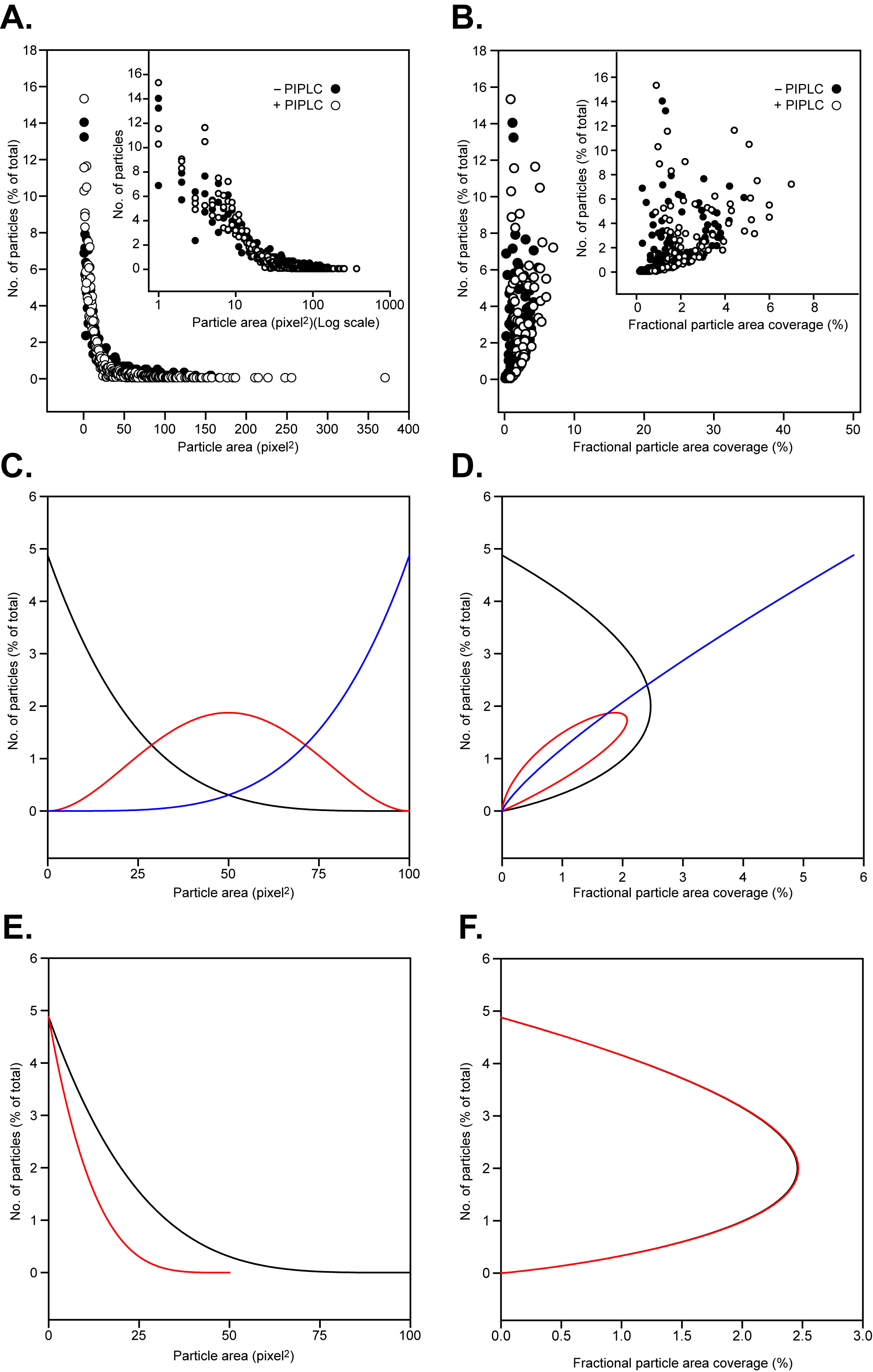

Supplement: Figure S2 — Particle analysis of PIN-α2δ cell surface clustering in the presence and absence of PI-PLC cell pre-treatment. A. Effect of PI-PLC on the size distribution of PIN-α2δ particles. Inset: data re-plotted using log scale. To facilitate overlay of images from separate cells, the number of particles Npi, of given area (Api, (abscissa) in pixel2) is expressed as a percentage of the total (Nt where Nt = Σ NAi). Note overlap in data, irrespective of pre-treatment with PI-PLC. B. Distribution of fractional coverage represented by PIN-α2δ particles. Inset: data re-plotted using expanded scale. Here and elsewhere [21], we define fractional coverage as the % of the total particulate area (Ct) within a region of interest (ROI), (not the area of the ROI) accounted for by particles of area Api (i.e. Npi.Api/Ct, where and Ap' is the area of the largest particle in the data set). Using this representation it is possible to discriminate cases where coverage of the total particle area arises from many small particles or a lesser number of larger particles. For example, in the simple situation where there are 4 particles each of size 10 pixel2 and 1 particle of size 60 pixel2, then Ct = 100, then for the smaller particles Npi/Nt = 0.8 and the fractional coverage = 0.4, for the larger particle Npi/Nt = 0.2 and fractional coverage = 0.6. In contrast, if the same total particulate area is comprised of 60 particles each of size 1 pixel2 and 4 particles each of size 10 pixel2, then Npi/Nt = 0.94 and the fractional coverage = 0.6, for the larger particles Npi/Nt = 0.06 and fractional coverage = 0.4). Note overlap of data, irrespective of pre-treatment with PI-PLC. Particle analysis was performed with Image J, using the adaptive thresholding plug-in, with thresholded images checked visually for accuracy. All data were extracted from 3 images from separate experiments. C. and D. Computer modelling of the effects of particle re-distribution on fractional coverage Fractional coverage graphs (D) w [file pone.0019802.s002.tif]

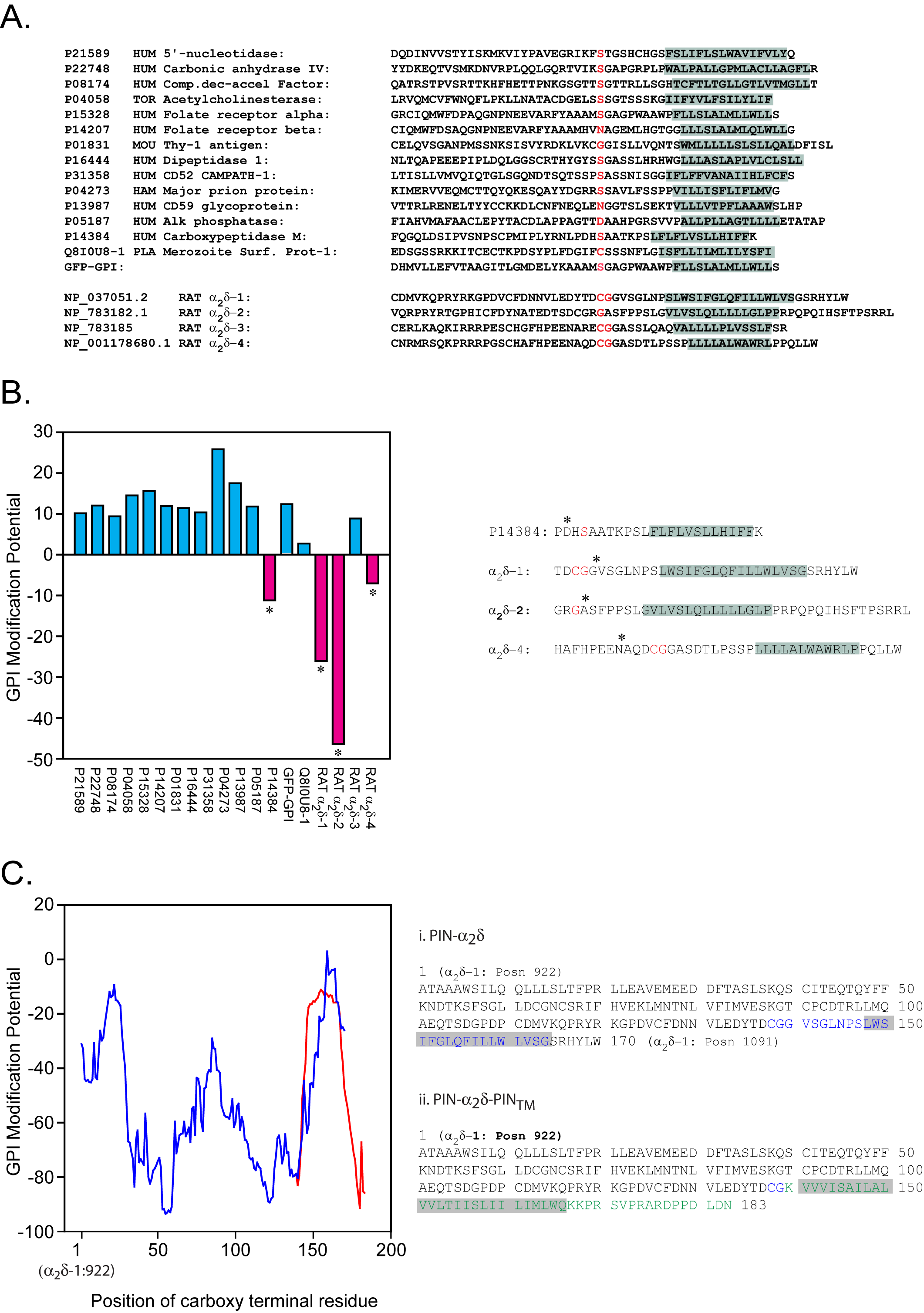

Supplement: Figure S3 — Comparative analysis of GPI-anchoring motifs. A. Comparison of carboxy-terminal sequences of known GPI-anchored proteins and rat α2δ-1,2,3 & 4 showing the ω site(s) (red lettering) and hydrophobic regions (grey boxes) of the GPI-anchoring motifs. All dataset examples (i.e. non-α2δ) correspond to proteins where the ω site has been verified, experimentally. (References: P21589: Misumi, Y. et al. (1990) Eur. J. Biochem. 191:563–569; P22748: Okuyama, T. et al. (1995) Arch. Biochem. Biophys. 320:315–322; P08174: Moran, P. et al. (1991) J. Biol. Chem. 266:1250–1257; P04058: Mehlert, A., et al. (1993) Biochem. J. 296:473–479; P15328 & P14207: Yan, W. and Ratnam, M. (1995) Biochemistry 34:14594–14600; P01831: Williams, A.F. and Gagnon, J. (1982) Science 216:696–703; P16444: Adachi, H. et al. (1990) J. Biol. Chem. 265:15341–15345; P31358: Xia, M.Q. et al. (1993) Biochem. J. 293:633–640; P04273: Stahl, N. et al. (1990) Biochemistry 29:8879–8884; P13987: Sugita, Y. et al. (1993) J. Biochem. 114:473–477; P05187: Micanovic, R. et al. (1990) Proc. Natl. Acad. Sci. U.S.A. 87:157–161; P14384: Tan, F. et al. (2003) Biochem. J. 370:567–578; XP_001352170.1: Hall, N. et al. (2002) Nature 419:527–531). The α2δ-1-3 ω sites have been tested, experimentally (Davies et al., 2010, Robinson et al., 2010 above), while that for α2δ-4 is inferred based on sequence homology to α2δ-3. B. Left panel: Potential for GPI-modification for dataset and α2δ proteins shown in A inferred using Big-Pi predictor software (http://expasy.org/tools/). Proteins with positive or negative GPI modification potential are shown in blue and red, respectively. Asterisks denote proteins where the ω site differs from that inferred. Right panel detailed sequence comparison of inferred (red lettering) and predicted (asterisks) ω sites. In most cases the inferred ω site is very close (<2 residues) to that found experimentally. C. Analysis of potential upstream GPI-anchoring motifs in the delta subunit of WT α2δ-1 (or PIN-α2δ [file pone.0019802.s003.tif]
