# Supplementary material for: Targeting of Voltage-Gated Calcium Channel α2δ-1 Subunit to Lipid Rafts Is Independent from a GPI-Anchoring Motif
Source: PLoS One. 2011 Jun 10;6(6):e19802. doi: 10.1371/journal.pone.0019802 (PMC3112168; doi:10.1371/journal.pone.0019802)
Supplement: Table S1 — Biophysical properties of Cav2.2/β1b channels co-expressed with WT α2δ-1, PIN-α2δ, PIN-α2δ-PINTMI and PIN-δ. I max is the maximum peak current density. Individual current density-voltage plots were fitted with a Boltzmann function:where V rev is the reversal potential, V 50,act is the voltage for half maximal activation of current, g is the conductance, and k is the slope factor. Statistical analysis used Students unpaired t-test. Asterisks denote statistically significant differences from (−)α2δ-1, as follows: * = P<0.05, *** = P<0.001. n is the number of cells tested per treatment. (DOC) [file pone.0019802.s004.doc]

| **Construct** | ***I*_max_**  **(pA.pF^-1^)** | ***V*_50_**  **(mV)** | ***k***  **(mV)** | **τ_act_**  **(ms)** | **τ_inact_**  **(ms)** | ***N*** |
| --- | --- | --- | --- | --- | --- | --- |
| (-)α_2_δ-1 | -16 ± 2 | 17 ± 1 | 5 ± 0.2 | 3.9 ± 0.2 | 536 ± 97 | 16 |
| α_2_δ-1 | -73 ± 11 ******* | 7 ± 0.4 ******* | 3 ± 0.2 ******* | 3.3 ± 0.2 | 274 ± 59 ***** | 13 |
| PIN-α_2_δ | -69 ± 11 ******* | 4 ± 1 ******* | 4 ± 0.3 ******* | 3.1 ± 0.3 | 298 ± 40 ***** | 12 |
| PIN-α_2_δ-PIN_TMI_ | -73 ± 13 ******* | 6 ± 1 ******* | 4 ± 0.2 ******* | 3.0 ± 0.1 ***** | 310 ± 41 ***** | 16 |
| PIN-δ | -13 ± 3 | 12 ± 2 ***** | 5 ± 0.2 | 4.1 ± 0.5 | 384 ± 70 | 13 |
